# Theta Burst Transcranial Magnetic Stimulation of Fronto-Parietal Networks: Modulation by Mental State

**DOI:** 10.20900/jpbs.20200011

**Published:** 2020-05-26

**Authors:** Stephan F. Taylor, Taraz G. Lee, John Jonides, Ivy F. Tso, Luis Hernandez-Garcia

**Affiliations:** 1 Departments of Psychiatry, University of Michigan, Ann Arbor, MI 48109, USA; 2 Departments of Psychology, University of Michigan, Ann Arbor, MI 48109, USA; 3 fMRI Laboratory, University of Michigan, Ann Arbor, MI 48109, USA

**Keywords:** dorsolateral prefrontal cortex, functional magnetic resonance imaging, cognitive control

## Abstract

Transcranial magnetic stimulation (TMS) treats neuropsychiatric disorders, but effects of stimulation are highly state-dependent and in most therapeutic applications, mental state is not controlled. This exploratory proposal will test the broad hypothesis that when TMS, specifically intermittent theta burst stimulation (iTBS), is applied during a controlled mental state, network changes will be facilitated, compared to stimulation when mental state is uncontrolled. We will focus on the dorsolateral prefrontal cortex (dlPFC) and the associated fronto-parietal network (FPN), which subserves cognitive control, an important neural and behavioral target of therapeutic TMS. After a baseline functional magnetic resonance imaging (fMRI) session, iTBS will be administered to 40 healthy subjects in three sessions over three days in a within-subjects, cross-over design: (1) dlPFC stimulation by iTBS alone, (2) dlPFC stimulation by iTBS while simultaneously performing a cognitive task, and (3) vertex (control) iTBS stimulation. Immediately after each iTBS session, we will measure blood oxygenation level-dependent (BOLD) activation during a cognitive control task (“*n*-back” task) and during the resting state, using BOLD connectivity and arterial spin labeling (ASL). We will test hypotheses that persisting neural changes and performance enhancement induced by iTBS to the dlPFC, compared to iTBS to the vertex, will affect the FPN, and these effects will be modulated by whether or not subjects receive iTBS when they are engaged in a cognitive control task. Demonstrating this interaction between iTBS and mental state will lay critical groundwork for future studies to show how controlling mental state during TMS can improve therapeutic effects.

**Trial Registration:**

Clinicaltrials.gov NCT04010461.

## INTRODUCTION

### The Need to Improve Our Understanding of Non-Invasive Brain Stimulation

Devices to deliver energy (magnetic, electrical, ultrasound) to nervous tissue have proliferated in the last decade, but there remains a dearth of knowledge about how these devices affect brain networks [1,2]. This proposal will focus on TMS, which uses magnetic energy to induce electrical currents in nervous tissue, and even though several decades of work has detailed effects of TMS on cortical physiology, many questions remain unanswered about how TMS affects the brain, particularly at the meso-scale of brain networks which subserve complex behaviors. TMS has frequently been employed to disrupt cortical activity with focal stimulation, inducing a “virtual lesion” and enabling inferences about the function of regions interrupted by stimulation [3,4], and it has also been used to enhance brain function [5,6]. In the United States, it has been cleared for therapeutic use in the treatment of major depressive disorder [7], obsessive-compulsive disorder [8] and migraine headache [9]. Numerous research studies have also reported benefits for many other neuropsychiatric conditions [10–15], and more recent work has sought to combine TMS with behavioral interventions [8,16–18]. While the combination of TMS with other interventions has intrinsic appeal, there is limited scientific value in showing that two therapies, combined, work better than a single therapy, alone. If these efforts are to be more than therapeutic mashups, we will need a deeper understanding of the effects of TMS on brain networks, as this R21 project proposes.

Our approach will focus on cognitive control and the underlying fronto-parietal networks (FPNs), which carry out this cognitive process. Cognitive control, also referred to as executive functioning, is the ability to flexibly adapt and regulate behavior in accord with goals and plans [19]. It is impaired in multiple neuropsychiatric conditions, such as depression [20], obsessive-compulsive disorder [21], dementia [22] and schizophrenia [23]. Fronto-parietal networks, comprised of bilateral, heteromodal cortex in the dorsolateral frontal convexity, connected with the parietal lobules via the longitudinal fasiculus, are engaged by tasks that require cognitive control [19,24,25]. Of relevance for therapeutic TMS, the dorsolateral prefrontal cortex, on the left and right hemispheres, is typically targeted by rTMS treatment for depression. As we review below, critical gaps in our knowledge exist about how TMS affects FPN and cognitive control, gaps which motivate the proposed work.

### What Is Known about How TMS Affects Brain Activity?

Non-repetitive pulses of TMS stimulate neurons and affect local microcircuitry in complex ways (see ref. [1,2]), but persisting, so-called “plastic”, effects require repetitive TMS (rTMS). The exact mechanism(s) of enduring changes in neural activity remain unknown. Persistent rTMS effects are complex, involving direct and indirect effects on excitatory and inhibitory neurons at the focus of stimulation, as well as secondary and tertiary effects on connected regions [26,27]. Timing, intensity, direction and frequency of stimulation all have differential effects [2,27,28]. In general, low frequency rTMS (≤1 Hz) reduces cortico-spinal excitability, measured as increasing levels of stimulation necessary to elicit a motor response (“motor evoked potential”, or MEP), whereas high frequency rTMS (generally ≥5 Hz) has an opposite, facilitatory effect on MEP, with effects generally lasting as long as the period of stimulation [2,28,29]. Longer lasting effects have been demonstrated by high frequency (50 Hz) bursts (“theta burst stimulation” or TBS), causing MEP facilitation for up to 60 minutes after a 190 s period of intermittent stimulation (iTBS). On the other hand, continuous TBS (cTBS) for 40 s causes MEP inhibition [30,31]. The TBS protocols were designed to elicit long term potentiation and long term depression, the most widely studied mechanisms of neural plasticity [32], although data suggests that responses measured in the MEP are more complex than LTP or LTD measured at the cellular level [33,34]. A variety of cellular and molecular effects of rTMS/TBS have been described (see [34] for recent review), and meso-scale effects have also been studied with electroencephalography [35–37], but in order to best localize network effects, this proposal will focus on the persisting effects revealed through neuroimaging, including resting perfusion/metabolism, task-related activation and connectivity.

In general, the most consistent neuroimaging result observed in “offline” (conducted after a period of rTMS stimulation) studies is that changes occur in regions beyond that stimulated, but anatomically and functionally connected [38–41]. Furthermore, it is not always possible to predict effects based on assumptions taken from MEP measurement. For example, “inhibitory” cTBS increased cerebral blood flow in the motor cortex in one study [42], and another study found that MEP measurement after “excitatory” iTBS exhibited an inverse relationship between BOLD signal and the MEP increase induced by iTBS [43]. cTBS to the dlPFC increased connectivity amongst regions of the FPN [44], and reduced the tuning of visual cortical activity and performance during a working memory task [45]. Overall, the effects of TMS on neuroimaging measures are difficult to predict and there are relatively few studies of offline effects of excitatory iTBS to the dlPFC. Therefore, Aim 1 of this proposal will address a question not sufficiently answered in the literature: What is the effect of iTBS to the FPN networks on resting perfusion, task-related activation and connectivity?

### State-Dependency of TMS Stimulation

A critical fact about TMS is that effects are highly state-dependent. The phenomenon known as “metaplasticity” refers to neural plasticity modulated by prior activity in a neuron [46], and an analogous process may occur with TMS [47,48]. Extracellular recordings in animals have shown that increased visual cortical activity during excitatory TMS leads to greater post-TMS activity [49]. From the earliest days of TMS research in humans, it was noted that stimulation of motor cortex during active muscle contraction increased the size and number of descending volleys compared to stimulation when the hand was at rest [50,51]. Since then, multiple examples of state-dependency have been described. For example, activating the ipsilateral hand during stimulation alters the response and coupling in the contralateral cortex during TMS to premotor cortex [52]. Directing attention to the contralateral hand during 5 Hz stimulation leads to larger MEP increases than when attention is directed to the ipsilateral hand [53]. Global changes in brain state, such as sleep, have demonstrated large effects, reducing the propagation of a single TMS pulse across the cortex when measured by surface electroencephalography [54]. In summary, multiple paradigms have demonstrated state-dependency of persisting TMS effects.

In spite of what is known about the state-dependency of TMS, most therapeutic uses of rTMS do not systematically control the mental state of subjects during stimulation, although this is starting to change. rTMS has been combined with psychotherapy for depression [16]. Cue exposure in addiction [55] and exposure therapy for post-traumatic stress disorder [17] and OCD [8] have been paired with TMS. A commercial system that combines rTMS with cognitive training in dementia patients has been cleared for marketing in the EU, even though differential effects in a shamcontrolled study were not found (*n* = 26) [18]. In spite of this work, there are no neuroimaging studies identifying the neural effects of state-dependency in paradigms relevant to therapeutic TMS. Examining motor function, Narayana and colleagues showed that, in a 4-week training paradigm, subjects who received 5 Hz rTMS to the motor cortex while they performed a digit sequence task showed improved motor performance and increased cerebral blood flow, relative to sham stimulation, in regions linked to skill learning [56]. For cognitive control, many studies have examined neural effects of TMS delivered “offline” (prior to neuroimaging) to a brain at rest [39,41,57,58], but virtually no work has examined offline, persisting effects of TMS delivered while a person is engaged in a task. These persisting effects of TMS interacting with brain state are critical to understand how this interaction could be harnessed for improved therapeutic effect. Thus, in Aim 2 we will address the question: How does mental state modulate the effect of rTMS on FPN networks?

The phenomenon of task state interacting with TMS has been used to target specific neural populations within a stimulated region, a paradigm known as “TMS adaptation” [59]. It has been used to augment brain mapping studies of language processing [60] and higher level perception [61]. For example, Silvanto and colleagues [62] showed that when subjects viewed visual motion while receiving inhibitory cTBS to direction-sensitive neurons in V1/V2, performance after stimulation was impaired for the direction-sensitive neurons that were not activated during the passive viewing task. In other words, neurons engaged by the task appeared to be “protected” from inhibitory cTBS. On the other hand, as we see below in the section on Preliminary Data, we have shown that iTBS during a memory task can improve working memory performance. These combined observations motivate our Aim 3, asking whether excitatory iTBS stimulation while the FPN is engaged would improve subsequent performance.

### Summary of Specific Aims

#### Specific Aim 1

Localize neural effects of dlPFC iTBS. We will show that persisting neural changes induced by iTBS to the dlPFC will affect the FPN. We predict that iTBS alone (subjects not performing a cognitive task), compared to vertex stimulation, will increase fMRI activation in the FPN during the n-back and increase FPN connectivity during resting state BOLD. We also predict that iTBS will increase resting perfusion in the FPN. Exploratory analyses will search for regions outside the FPN that change with stimulation to develop a comprehensive picture of how iTBS to the dlPFC interacts with brain networks.

#### Specific Aim 2

Demonstrate modulation of the effect of dlPFC iTBS by a cognitive task. We predict that FPN changes when iTBS is administered while subjects perform the n-back task will be greater than when they are not performing a task. We will also test the same hypothesis for BOLD resting state connectivity, and ASL-measured perfusion, in addition to exploratory hypotheses on brain networks outside the FPN.

#### Specific Aim 3

Demonstrate improvement in cognitive control with iTBS, modulated by cognitive task during stimulation. We will test predictions that n-back performance will improve following iTBS to dlPFC, but not vertex, and will improve even more following iTBS during n-back performance. Exploratory analyses will examine correlations between performance changes and network changes, suggesting mechanistic connections between iTBS stimulation and performance changes.

## INNOVATION

### Three-pronged neuroimaging study of TMS effects

This exploratory R21 proposal will provide a broad assessment to anatomically localize TMS effects on the FPN(s), using three complementary neuroimaging measurements: (1) BOLD activation, (2) resting state functional connectivity and (3) quantitative cerebral blood flow (CBF). Quantitative CBF will be measured with arterial spin labeling (ASL) fMRI. Unlike typical fMRI studies with blood oxygen-level dependent (BOLD) fMRI, which provides unitless measures of change across short time frames (generally <30 s), ASL fMRI yields an absolute measure of cerebral blood flow [63,64], which is highly correlated with more direct measures of cellular metabolism [65,66], less susceptible to signal drift than BOLD [67], without significant susceptibility artifact [68] and with greater stability over time [69,70]. The combination of three measures will permit us to examine multiple levels of TMS effects: dynamic (activation), static (perfusion) and network (resting state connectivity). For example, a failure to find changes in BOLD activation after stimulation might reflect an increase in baseline CBF, and we will be able to address that possibility. With a large degree of uncertainty about where TMS effects occur, it is critical to have a broad experimental scope as we propose here.

## APPROACH

### Preliminary Data: Enhancing Memory Function

When TMS was initially applied to individuals performing tasks, researchers assumed that it would disrupt function, although it has since been demonstrated multiple times that TMS can improve performance in a variety of tasks [5,6]. With online stimulation, we have demonstrated improvement in verbal list learning [71]. A brief TBS (2 s) train was delivered just prior to the presentation of a list words, about which subjects made a semantic judgment to encourage deep encoding. In a subsequent memory test, subjects had better recall of words when they were stimulated at the left dlPFC, compared to stimulation at the vertex. Of note, 5 different delays between TBS pulse and the beginning of the word presentation occurred (700 ms, 5 s, 7 s, 11 s, 15 s), showing that improved learning occurred for up to 15 s after TBS. This is consistent with the persistent effects noted with iTBS [30,31]. Although the studies of offline effects of TMS on working memory have been mixed, with positive [72] and negative [73,74] results, a recent meta-regression of regular patterned, high frequency rTMS (≥5 Hz) to the dlPFC showed that offline stimulation significantly improved reaction time and accuracy in *n*-back tasks [75]. Taken together, these data demonstrate: (1) our own capacity to perform iTBS studies, and (2) evidence that TMS and iTBS can potentiate circuitry to improve a cognitive control.

## DESIGN

### Overview

**Figure 1.**
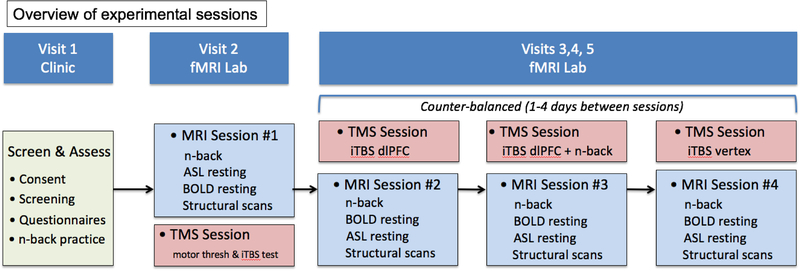
Experimental design.

We propose a within-subjects design that will expose 40 subjects to three TMS sessions, with each followed by an MRI session. The design is depicted in Figure 1. The first MRI session, without TMS, will obtain baseline measurements of FPN activation (providing the target for iTBS stimulation—see below), resting state connectivity and resting state cerebral perfusion. The TMS sessions, separated by 1–4 days, will provide the experimental manipulation, wherein subjects will receive iTBS to the dlPFC, while they simultaneously perform an *n*-back working memory task, or while they are in an unconstrained, resting state. A third iTBS session will be delivered to the vertex when subjects are in resting state, serving as a control condition for dlPFC stimulation, as in our pilot study [71]. Although some protocols use either sham stimulation or a 45 degree tilt of the TMS coil to provide a control for stimulating a desired target, it is important to control for the strong somatic sensations of receiving the TMS stimulus, which neither sham nor 45-degree tilt do, in order to show regional specificity of stimulation to the dlPFC. There is also evidence that the 45-degree tilt excites neural tissue [76]. The order of the three TBS sessions will be counter-balanced across subjects to mitigate practice effects in the *n*-back. We considered adding a session with vertex stimulation and an *n*-back task, but given the demands that four MRI sessions already place on participants, and the contrast with vertex stimulation + task was least valuable for our Aims, we elected to skip a 5th MRI session.

### Subjects, Screening and Assessment

Participants will be healthy individuals, ages 18–50. The upper limit of 50 will reduce functional anatomic variance from older subjects, who exhibit less specific activation patterns [77,78]. We will recruit ~48 individuals (50% women) to allow for drop-outs and subjects who cannot tolerate TBS. Screening by the M.I.N.I. [79]. will exclude subjects with a current or past history of mental illness (simple phobias allowed) or past or current substance use disorders within the last 5 years. Potential participants will have a complete medical review of systems, in addition to being screened with the Transcranial Magnetic Stimulation Adult Safety Screen [80] form in order to exclude participants with contraindications for TMS and MRI, such as: a previous adverse reaction to TMS, history of seizures or a condition that would increase the likelihood for seizures, family history of epilepsy, taking medications that would increase the risk of seizure, pregnant or trying to become pregnant, presence of metal in the head (other than in the mouth), claustrophobia, presence of an implanted device like a pacemaker, metal in the brain, other brain injuries or brain-related conditions.

The assessment session will also include acquisition of basic demographic data, as well as completion of psychological scales to be used in post-hoc analyses, including the Beck Depression Inventory II [81] to assess sub-clinical mood symptoms, Short Form 12 [82] to assess general health status, the reading portion of the Wide-Range Achievement Test [83]. to assess general educational attainment. To assess executive function, we will administer the Trail Making Test, part B (TMT) [84]. To assess working memory, we will also administer the digit span task [85]. In the assessment session, subjects will practice the *n*-back task inside a mock scanner in order to familiarize them with this task and reduce subsequent practice effects across sessions. Practice effects are expected to be minimal after the initial exposure to the *n*-back. We have found in a 2-back task, after initial practice, subjects (*n* = 32) exhibited only a 2.1 ms (±94 S.D.) drop in RT and a 0.9% (±0.27 S.D.) improvement in d-prime accuracy when tested in a subsequent session [86]. The last step in the assessment session will be a trial run of iTBS, beginning with motor threshold determination and then exposing subjects to a ~3 min run of iTBS.

The initial safety assessment from the screening visit (Visit 1) will be repeated before each MRI session (Visits 2–5). We will review the subject’s safety screening forms for fMRI and TMS to make sure nothing has changed since the initial assessment, and we will also record sleep and ingestions of psychoactive substances (caffeine, alcohol, nicotine). At screening, we will remind subjects to get a good night’s sleep and abstain from alcohol and drugs on the evenings before each MRI session. We will make every effort to schedule the 3 iTBS sessions at the same time of day, avoiding Mondays. Overall, the intention is to reduce variability in the subject’s state due to exogenous factors, and to ensure that they continue to meet safety criteria for the TMS procedure.

### Description of Cognitive Control “*N*-Back” Task

To elicit cognitive control and the associated FPN, we will use the “*n-*back” task, a widely studied and highly robust activator of FPN, which, unlike many other tasks tapping cognitive control, can provide significant activation patterns at the level of the individual subject [87–89]. Generating reliable activation patterns for individual subject will allow us to localize individually-specific activation patterns for targeting TMS stimulation. The task has multiple components, including short-term storage, information manipulation and recognition. Subjects will see a string of letters, presented for 0.5 s, every 2 s. The target condition will test for 3-back recall, in which subjects respond with a button press to a target that matches the letter from 3 letters before. This will be contrasted with a 1-back condition, in which subjects will respond to a letter that matches the letter previously presented. While some investigators use a “0-back” control condition, wherein subjects respond to a letter matching a single target letter, that variation on the n-back design does not truly isolate cognitive-control processes. The 1-back control condition requires a simple working memory storage, which must be updated with every stimulus, unlike the O-back task, which does not require updating in working memory. Blocks will run for 20 s, preceded by an instruction to respond “3-back” or “1-back”. Blocks will be separated by a 12 s baseline (eyes on a fixation cross), and subjects will do 2 runs for a total of 10 min.

### MRI Acquisitions

Scanning will be performed at the University of Michigan fMRI Laboratory on a 3T GE MR 750 scanner (Waukesha, WI) using a 32-channel coil. For the structural and BOLD acquisitions, we will leverage the UM experience with the Adolescent Brain and Cognitive Development study, ensuring state-of-the-art, high resolution data with multi-band acquisition. After localizer scans, subjects will undergo 3 scans, which will occur within 40 minutes of the subject leaving the TMS suite: (1) BOLD-weighted fMRI (EPI, TR = 800 ms, FOV = 23, slice = 2.4 mm, 96 × 96 matrix) for the *n-*back task; (2) BOLD-weighted resting state scan for 10 min to obtain data for functional connectivity analyses (eyes open, looking at fixation cross); (3) pseudo-continuous ASL (pCASL) sequence following the recommendations of the ISMRM consensus [90]. Our pCASL implementation will use a 3D stack of spirals acquisition [91] with 6 interleaves (TR = 5000 ms, TE = 4 ms, BW = 125 kHz, FOV = 22 cm) preceded by a 1800 ms labeling period and an 1800 ms delay. Two hyperbolic secant inversion pulses at 600 and 1300 ms after labeling will suppress background signals, after first 2 frames to permit collection of spin density images for quantification. We will acquire 20 pCASL control-label image pairs, reconstructed to a 128 × 128 matrix. Since the excitatory effects of iTBS have been demonstrated to last up to 60 min [30], data acquisitions will occur in the appropriate time frame to capture iTBS effects. The last scan in the sequence will be a high-resolution structural scan (SPGR) for image normalization, as well as a T1 scan in the same prescription as the BOLD scans to facilitate normalization.

### TMS Procedure

TMS will be delivered through a MagPro X100 with MagOption magnetic stimulator and a 90 mm figure-8 coil (MC-B70, MagVenture Inc.). MEP elicited using biphasic posterior-anterior stimulation and coil oriented 45 degree to coronal plane will be recorded from right first dorsal interosseous (FDI) using surface electromyography (Rogue Research, Montreal). Active motor threshold (AMT), measured at the first MRI session (Visit 2), will be obtained as the percentage of stimulator output that elicits an MEP of ≥50 μV peak-to-peak on ten out of twenty trials while the subject is contracting the FDI muscle at approximately 20% of maximum [92]. Immediately prior to entering the MRI scanner, for visits 3 through 5, we will deliver iTBS [30], using 3 pulses of stimulation at 50 Hz, repeated every 200 ms, for 2 s trains, repeated every 10 s, for a total of 600 pulses in 190 s. Stimulation will be delivered at 80% of MT, within consensus recommendations for safety [93].

To determine the site for stimulation, we will use neuronavigation with structural and functional information to account for individual variability in recruitment of the FPN. For the dlPFC, we will locate the junction of the anterior and middle third of the left middle frontal gyrus (MFG), which corresponds to Brodman area 46 [94,95], and within a zone that is 1/3 the length of the MFG, we will search for the locus of greatest activation in BOLD activation (3-back minus 1-back) for each subject, using those coordinates as the site of stimulation. The Brainsight Frameless system (Rogue Research, Montreal CA) will align structural and functional images to identify the overlaying scalp position. For the vertex (control) target, we will locate, in the vicinity of the vertex defined by the 10–20 system, the area of minimal activation to the *n*-back task. Online monitoring with the Brainsight system will allow the TMS operator to maintain precise positioning of the coil over the target.

For vertex stimulation and one of the dlPFC stimulation sessions, subjects will be instructed to maintain gaze on a fixation screen and rest. For dlPFC stimulation augmented with a task, the screen will display an *n-*back task, using the 3-back condition. Letters will appear for 0.5 s every 2 s, and presentation will be synchronized with the TBS stimulation, which will occur 0.5 s before the onset of every 5th letter.

### General Analysis Plan

MRI data analysis and strict quality control will use validated and established routines implemented in our laboratory. For processing BOLD images, we will use well-tested routines from FSL 5.0.1 and SPM12 for slice timing and realignment algorithms and spatial normalization (MNI-152) procedures implemented in VBM8. First-level analysis will use the general framework of the modified General Linear Model [96], implemented in SPM12 with temporal convolution. For the *n-back activation* task, the first-level design matrix will include regressors for 3-back and 1-back, MRI/TBS session and 12 realignment parameters (including temporal derivatives of 6 realignment parameters). Second-level, between-subject analysis will consist of two-sided, one-sample *t*-tests for primary effects. For statistical inference across the FPN, and the entire brain, we will use topological false discovery rate (FDR) [97] providing corrected probabilities at *p* < 0.017 (0.05/3, accounting for testing of 3 MRI measures). Given *a priori* hypotheses about FPN, a mask of activation (3-back minus 1-back, *p* < 0.001_uncorr_) at the group level will be used as small volume correction. We will conduct exploratory analyses with a generalized psychophysical interaction (gPPI), to test for interaction between task and session, extracting the time-course at the site of stimulation, deconvolving it with the canonical hemodynamic response function (HRF), and then generating an interaction regressor (task × time-course) which is tested (after-reconvolution with HRF) across the entire brain [98]. Statistical correction will be as above.

For the *functional connectivity analysis,* multiple regression will be applied to normalized, realigned, 4-D image sets to remove nuisance variables (24 movement regressors, 5 white matter & CSF PCA components [99], band pass filtered 0.01–0.1 Hz, motion scrubbed [100] and de-noised with ICA-AROMA [101]). For the primary analysis, the time-course from a 6 mm radius sphere at the site of stimulation will be entered into the multiple regression and correlated with every other gray-matter voxel time-course in the brain for each subject. Correlation coefficients will be Z-transformed and then entered into the second level, between-subject analyses as two-sided, paired *t*-tests to compare between sessions. Statistical inference will be controlled as above. Exploratory analyses will examine a graph-theoretic measure (global brain connectivity) [102], and amplitude of low frequency fluctuations [103] to fully understand TBS effects.

The *pCASL images* will be realigned using SPM12 realignment routines and denoised using compCor [99] and then used to calculate the perfusion rate at each voxel using a two-compartment model, assuming gray matter T1 of 1400 ms. and 90% labeling efficiency. ROI analysis of the CBF images will occur in native space, using the FPN mask derived from *n*-back activation. A two-sided, paired *t*-test will test for effect of sessions, with *p* < 0.017 as threshold. Secondary analysis will be performed on a 6 cm radius sphere at the site of stimulation, in addition to a whole brain, voxel-wise search for changes in gray-matter tissue, using topological FDR to correct for multiple comparisons to *p* < 0.017.

For analysis of n-back performance, our primary performance measure will be d-prime, to capture the sensitivity to the target and not bias to respond. A secondary measure will be reaction time(RT) to the 3-back condition, shown to be sensitive to TMS stimulation in a published study [72]. For each measure, we will conduct a repeated measures ANCOVA, with the performance measure across the three TBS conditions as the repeated measure, and sex as a fixed, between subject factor and age as a co-variate. Post-hoc, paired *t*-tests will used for specific contrasts.

### Analysis of Specific Aims

#### Aim 1 analysis

To examine the effect of iTBS on FPN, we will directly contrast the MRI session following dlPFC stimulation with the session following vertex stimulation, with subjects in an unconstrained, resting state for both conditions. We predict that iTBS to dlPFC, compared to vertex stimulation, will (H1.1) increase fMRI activation in the FPN during the *n*-back, (H1.2) increase FPN connectivity during resting state BOLD, and (H1.3) increase resting perfusion at the site of stimulation. Because TMS effects are observed away from the site of stimulation [38–41], we are testing functionally-defined FPN networks, defined both by n-back activation and connectivity. Exploratory analyses will search for regions outside the FPN that change with stimulation to develop a comprehensive picture of how iTBS interacts with brain networks. For example, dlPFC stimulation has been observed to induce changes in a limbic anterior-cingulate network [104]. More intriguingly, a single session of iTBS to the dlPFC in healthy subjects has been shown to reduce default mode connectivity at the rostral-dorsal anterior cingulate gyrus [105], a finding we will seek to replicate. We are using two-sided tests, because unpredicted results have been described in the literature, where inhibitory cTBS has resulted in increased perfusion [42], and increased connectivity [44], and excitatory 5 Hz TMS to dlPFC demonstrated reduced BOLD activation [106]. Comparing multiple MRI measures will be important. For example, if resting perfusion is increased, BOLD activation may decrease because of a ceiling effect of increased excitation. Exploratory analyses will also test for correlations between the three measures, to provide a richer picture of TBS effects. Lastly, we recognize that vertex stimulation, selected to avoid the FPN and serve as a control condition for dlPFC stimulation, may have some effects on brain networks. With some caveats (effect of order, since baseline is not counter-balanced), we can use baseline measures from the first MRI session (no preceding TMS session), to see if TBS differences represent a change in the dlPFCstimulated condition or the vertex-stimulated condition.

#### Aim 2 analysis

To examine state-dependency and modulation of iTBS effects by task, we will contrast MRI measures following iTBS applied to the dlPFC while subjects perform the n-back with MRI measures following iTBS compared to when they are in an unconstrained, resting state. We will test the following: (H2.1) FPN activation to the n-back task will be greater than when they are not performing a task; (H2.2) connectivity with the dlPFC site of stimulation will increase, and (H2.3) resting perfusion at stimulation site will increase. Although the directions of change are difficult to predict, we are predicting increasing activity as has been reported for iTBS applied during a deep encoding memory paradigm and not observed during a shallow-encoding [107]. However, it is possible that cortex may become more efficient and less activation may be seen.

#### Aim 3 analysis

To examine the effects of mental state on performance, we will test the hypothesis that (H3.1) d-prime accuracy will be greater for dlPFC stimulation (no task during iTBS) relative to vertex stimulation, and (H3.2) d-prime accuracy will be greater for dlPFC stimulation applied while subjects carry out an *n*-back during iTBS stimulation during stimulation, compared to when they receive stimulation alone. The same hypotheses will be applied to median RT, our secondary measure. Twosided tests will be used, because it is also possible that performance may worsen, either with stimulation alone, or when combined with subjects performing the *n*-back. It would be critical to know, for example, that performance of a cognitive control task was degraded by combining TMS with a task. Although a commercial device is being tested on dementia patients, relying on the assumption that the combination is beneficial [18], we are not aware of this assumption being tested as we propose to do here. In addition to testing questions about performance, we will also examine correlations between performance and the MRI measures from Aims 1 and 2, testing important questions around mechanism.

#### Sex, age, potential problems and limitations

Subject variance may affect results. We will perform post-hoc tests on sex and age as effects. Our behavioral analysis will enter these two factors into the analysis. Other post-hoc tests will explore the impact of sub-clinical mood symptoms (BDI), executive function (TMT & digit span) and general health status (SF-12) on observed effects. For example, effects may only be observed in subjects with relatively low levels of executive function, so population-normed tests will help to assess this possibility. Although the literature suggests that the MEP effects of iTBS last for up to 60 min [30], the effects on perfusion, connectivity and task-induced activation may have different dynamics. Exploring possible temporal differences in these effects would be an important follow-up study to perform in order to better characterize the effects of time on iTBS-induced neural effects. Another related limitation to consider is that the order of our scan acquisitions is designed to be identical for all subjects, largely to avoid variance associated with these potential dynamic changes in the effects of iTBS. However, this choice to reduce variance entails order effects in the scanning sequence, e.g., task activation affecting the resting state signal measured after task performance [108].

#### Power analysis

Our within-subjects design, with 40 subjects, will provide sufficient experimental power. The n-back task employs a robust block design with large effect sizes (ES, range of 0.8 to 2.6) and good within-subject reliability (ICC 0.44–0.65) in *a priori* defined regions, according to Plichta et al. [109]. We will have double the number of cycles (10) as Plichta et al., placing this design very high in relative power of block designs [110]. The general form of the analysis will consist of paired *t*-tests, and with 40 subjects, we will be able to detect moderate effect sizes of 0.53 (α = 0.017, two-tailed) at 80% power. Primary analyses for Aims 1 and 2 will harness the power of voxel-wise analysis in defined FPNs. Even with a relatively insensitive, *a priori* defined FPN [86], we would be able to detect a 19% change in connectivity for the entire FPN (80% power, α = 0.017, two-tailed). For the pCASL analysis, we take test-retest reliability from Chen et al. [111], yielding a coefficient of variation of 8.5% for average gray matter, which means that with 40 subjects, we will be able to detect a 4.42% difference in perfusion at a power of 80% (α = 0.017, two-tailed). For Aim 3, taking data from a 2-back task conducted in depressed patients, tested over two sessions [86] and assuming similar variance, we should be able to detect a 49 msec change in RT, or a 1.4% change in d-prime accuracy, at a power of 80% (α = 0.017, two-tailed). This detectable change in RT is approximately half of what was reported in the literature for 5 Hz TMS to the dlPFC by one group [72], and well below the approximately 20% improvement in d-prime we reported in our preliminary data [71]. Overall, our experimental power in this exploratory R21 should be more than sufficient to address our study aims.

## CONCLUSION: IMPACT AND FUTURE DIRECTIONS

Expected results from this study will provide the first proof-of-concept that modulating mental state with a cognitive control task during TMS will identify network(s) associated with this effect, showing where and how it occurs in the brain. By showing the neurocircuit changes associated with iTBS, demonstrating how task-engagement of FPN will change these effects on the network and showing more behavioral improvement when iTBS is matched with a cognitive task, we will take a first critical step toward *target engagement, albeit in non-ill subjects. The results will provide* a mechanistic foundation for studies of therapeutic TMS, enabling one to show that combination treatment is more than a therapeutic mashup. The next step will be an R61/R33, first seeking to show *target engagement* in depressed patients, employing multiple sessions and higher stimulation intensity [112], followed by a study to show that manipulating mental state during therapeutic TMS will have beneficial effects for the treatment of depression. It is also possible that performance after iTBS during a cognitive task could worsen performance on that task, a finding which would provide strong evidence for what not to have patients do while they are receiving TMS treatment. In either case, results can be translated to TMS interventions for dementia or any other condition where cognitive control is impaired and needs to be enhanced.

## References

[1] Volz LJ, Hamada M, Rothwell JC, Grefkes C. What Makes the Muscle Twitch: Motor System Connectivity and TMS-Induced Activity. Cereb Cortex. 2015;25(9):2346–53. 24610120 10.1093/cercor/bhu032

[2] Fitzgerald PB, Fountain S, Daskalakis ZJ. A comprehensive review of the effects of rTMS on motor cortical excitability and inhibition. Clin Neurophysiol. 2006;117(12):2584–96. 16890483 10.1016/j.clinph.2006.06.712

[3] Paus T Inferring causality in brain images: a perturbation approach. Philos Trans R Soc Lond B Biol Sci. 2005;360(1457):1109–14. 16087451 10.1098/rstb.2005.1652 PMC1854935

[4] Wagner T, Rushmore J, Eden U, Valero-Cabre A. Biophysical foundations underlying TMS: setting the stage for an effective use of neurostimulation in the cognitive neurosciences. Cortex. 2009;45(9):1025–34. 19027896 10.1016/j.cortex.2008.10.002 PMC3417820

[5] Guse B, Falkai P, Wobrock T. Cognitive effects of high-frequency repetitive transcranial magnetic stimulation: a systematic review. J Neural Transm (Vienna). 2010;117(1):105–22. 19859782 10.1007/s00702-009-0333-7 PMC3085788

[6] Luber B, Lisanby SH. Enhancement of human cognitive performance using transcranial magnetic stimulation (TMS). NeuroImage. 2014;85(Pt 3):961–70. 23770409 10.1016/j.neuroimage.2013.06.007 PMC4083569

[7] McClintock SM, Reti IM, Carpenter LL, McDonald WM, Dubin M, Taylor SF, Consensus Recommendations for the Clinical Application of Repetitive Transcranial Magnetic Stimulation (rTMS) in the Treatment of Depression. J Clin Psychiatry. 2018;79(1):16cs10905. doi: 10.4088/JCP.16cs10905 PMC5846193 28541649

[8] Carmi L, Alyagon U, Barnea-Ygael N, Zohar J, Dar R, Zangen A. Clinical and electrophysiological outcomes of deep TMS over the medial prefrontal and anterior cingulate cortices in OCD patients. Brain Stimul. 2018;11(1):158–65. 28927961 10.1016/j.brs.2017.09.004

[9] Lan L, Zhang X, Li X, Rong X, Peng Y. The efficacy of transcranial magnetic stimulation on migraine: a meta-analysis of randomized controlled trails. J Headache Pain. 2017;18(1):86. 28831756 10.1186/s10194-017-0792-4 PMC5567575

[10] Gonsalvez I, Baror R, Fried P, Santarnecchi E, Pascual-Leone A. Therapeutic Noninvasive Brain Stimulation in Alzheimer’s Disease. Curr Alzheimer Res. 2017;14(4):362–76. 27697061 10.2174/1567205013666160930113907

[11] Trevizol AP, Barros MD, Silva PO, Osuch E, Cordeiro Q, Shiozawa P. Transcranial magnetic stimulation for posttraumatic stress disorder: an updated systematic review and meta-analysis. Trends Psychiatry Psychother. 2016;38(1):50–5. 27074341 10.1590/2237-6089-2015-0072

[12] Klein MM, Treister R, Raij T, Pascual-Leone A, Park L, Nurmikko T, Transcranial magnetic stimulation of the brain: guidelines for pain treatment research. Pain. 2015;156(9):1601–14. 25919472 10.1097/j.pain.0000000000000210 PMC4545735

[13] Shah-Basak PP, Wurzman R, Purcell JB, Gervits F, Hamilton R. Fields or flows? A comparative metaanalysis of transcranial magnetic and direct current stimulation to treat post-stroke aphasia. Restor Neurol Neurosci. 2016;34(4):537–58. 27163249 10.3233/RNN-150616

[14] Berardelli A, Suppa A. Noninvasive brain stimulation in Huntingtonʼs disease. Handb Clin Neurol. 2013;116:555–60. 24112923 10.1016/B978-0-444-53497-2.00044-9

[15] Fitzgerald PB, Daskalakis ZJ. A review of repetitive transcranial magnetic stimulation use in the treatment of schizophrenia. Can J Psychiatry. 2008;53(9):567–76. 18801219 10.1177/070674370805300903

[16] Donse L, Padberg F, Sack AT, Rush AJ, Arns M. Simultaneous rTMS and psychotherapy in major depressive disorder: Clinical outcomes and predictors from a large naturalistic study. Brain Stimul. 2018;11(2):337–45. 29174304 10.1016/j.brs.2017.11.004

[17] Kozel FA, Motes MA, Didehbani N, DeLaRosa B, Bass C, Schraufnagel CD, Repetitive TMS to augment cognitive processing therapy in combat veterans of recent conflicts with PTSD: A randomized clinical trial. J Affect Disord. 2018;229:506–14. 29351885 10.1016/j.jad.2017.12.046

[18] Lee J, Choi BH, Oh E, Sohn EH, Lee AY. Treatment of Alzheimerʼs Disease with Repetitive Transcranial Magnetic Stimulation Combined with Cognitive Training: A Prospective, Randomized, Double-Blind, Placebo-Controlled Study. J Clin Neurol. 2016;12(1):57–64. 26365021 10.3988/jcn.2016.12.1.57 PMC4712287

[19] Miller EK, Cohen JD. An integrative theory of prefrontal cortex function. Annu Rev Neurosci. 2001;24:167–202. 11283309 10.1146/annurev.neuro.24.1.167

[20] Snyder HR. Major depressive disorder is associated with broad impairments on neuropsychological measures of executive function: a meta-analysis and review. Psychol Bull. 2013;139(1):81–132. 22642228 10.1037/a0028727 PMC3436964

[21] Delorme R, Gousse V, Roy I, Trandafir A, Mathieu F, Mouren-Simeoni MC, Shared executive dysfunctions in unaffected relatives of patients with autism and obsessive-compulsive disorder. Eur Psychiatry. 2007;22(1):32–8. 17127035 10.1016/j.eurpsy.2006.05.002 PMC1894853

[22] Mowszowski L, Lampit A, Walton CC, Naismith SL. Strategy-Based Cognitive Training for Improving Executive Functions in Older Adults: a Systematic Review. Neuropsychol Rev. 2016;26(3):252–70. 27613718 10.1007/s11065-016-9329-x

[23] Lesh TA, Niendam TA, Minzenberg MJ, Carter CS. Cognitive control deficits in schizophrenia: mechanisms and meaning. Neuropsychopharmacology. 2011;36(1):316–38. 20844478 10.1038/npp.2010.156 PMC3052853

[24] Badre D Cognitive control, hierarchy, and the rostro-caudal organization of the frontal lobes. Trends Cogn Sci. 2008;12(5):193–200. 18403252 10.1016/j.tics.2008.02.004

[25] Braver TS. The variable nature of cognitive control: a dual mechanisms framework. Trends in cognitive sciences. 2012;16(2):106–13. 22245618 10.1016/j.tics.2011.12.010 PMC3289517

[26] Driver J, Blankenburg F, Bestmann S, Vanduffel W, Ruff CC. Concurrent brainstimulation and neuroimaging for studies of cognition. Trends Cogn Sci. 2009;13(7):319–27. 19540793 10.1016/j.tics.2009.04.007

[27] Bestmann S The physiological basis of transcranial magnetic stimulation. Trends Cogn Sci. 2008;12(3):81–3. 18243042 10.1016/j.tics.2007.12.002

[28] Pascual-Leone A, Tormos JM, Keenan J, Tarazona F, Canete C, Catala MD. Study and modulation of human cortical excitability with transcranial magnetic stimulation. J Clin Neurophysiol. 1998;15(4):333–43. 9736467 10.1097/00004691-199807000-00005

[29] Siebner HR, Rothwell J. Transcranial magnetic stimulation: new insights into representational cortical plasticity. Exp Brain Res. 2003;148(1):1–16. 12478392 10.1007/s00221-002-1234-2

[30] Wischnewski M, Schutter DJ. Efficacy and Time Course of Theta Burst Stimulation in Healthy Humans. Brain Stimul. 2015;8(4):685–92. 26014214 10.1016/j.brs.2015.03.004

[31] Huang YZ, Edwards MJ, Rounis E, Bhatia KP, Rothwell JC. Theta burst stimulation of the human motor cortex. Neuron. 2005;45(2):201–6. 15664172 10.1016/j.neuron.2004.12.033

[32] Bear MF, Malenka RC. Synaptic plasticity: LTP and LTD. Curr Opin Neurobiol. 1994;4(3):389–99. 7919934 10.1016/0959-4388(94)90101-5

[33] Ziemann U, Paulus W, Nitsche MA, Pascual-Leone A, Byblow WD, Berardelli A, Consensus: Motor cortex plasticity protocols. Brain Stimul. 2008;1(3):164–82. 20633383 10.1016/j.brs.2008.06.006

[34] Cirillo G, Di Pino G, Capone F, Ranieri F, Florio L, Todisco V, Neurobiological after-effects of non-invasive brain stimulation. Brain Stimul. 2017;10(1):1–18. 27931886 10.1016/j.brs.2016.11.009

[35] Thut G, Pascual-Leone A. A review of combined TMS-EEG studies to characterize lasting effects of repetitive TMS and assess their usefulness in cognitive and clinical neuroscience. Brain Topogr. 2010;22(4):219–32. 19862614 10.1007/s10548-009-0115-4 PMC3260526

[36] Hill AT, Rogasch NC, Fitzgerald PB, Hoy KE. TMS-EEG: A window into the neurophysiological effects of transcranial electrical stimulation in non-motor brain regions. Neurosci Biobehav Rev. 2016;64:175–84. 26959337 10.1016/j.neubiorev.2016.03.006

[37] Daskalakis ZJ, Farzan F, Barr MS, Maller JJ, Chen R, Fitzgerald PB. Longinterval cortical inhibition from the dorsolateral prefrontal cortex: a TMS-EEG study. Neuropsychopharmacology. 2008;33(12):2860–9. 18322469 10.1038/npp.2008.22

[38] Bergmann TO, Karabanov A, Hartwigsen G, Thielscher A, Siebner HR. Combining non-invasive transcranial brain stimulation with neuroimaging and electrophysiology: Current approaches and future perspectives. Neuroimage. 2016;140:4–19. 26883069 10.1016/j.neuroimage.2016.02.012

[39] Siebner HR, Bergmann TO, Bestmann S, Massimini M, Johansen-Berg H, Mochizuki H, Consensus paper: combining transcranial stimulation with neuroimaging. Brain Stimul. 2009;2(2):58–80. 20633405 10.1016/j.brs.2008.11.002

[40] Fox MD, Halko MA, Eldaief MC, Pascual-Leone A. Measuring and manipulating brain connectivity with resting state functional connectivity magnetic resonance imaging (fcMRI) and transcranial magnetic stimulation (TMS). Neuroimage. 2012;62(4):2232–43. 22465297 10.1016/j.neuroimage.2012.03.035 PMC3518426

[41] Stagg CJ, O’Shea J, Johansen-Berg H. Imaging the effects of rTMS-induced cortical plasticity. Restor Neurol Neurosci. 2010;28(4):425–36. 20714067 10.3233/RNN-2010-0553

[42] Orosz A, Jann K, Wirth M, Wiest R, Dierks T, Federspiel A. Theta burst TMS increases cerebral blood flow in the primary motor cortex during motor performance as assessed by arterial spin labeling (ASL). Neuroimage. 2012;61(3):599–605. 22613775 10.1016/j.neuroimage.2012.03.084

[43] Cardenas-Morales L, Volz LJ, Michely J, Rehme AK, Pool EM, Nettekoven C, Network connectivity and individual responses to brain stimulation in the human motor system. Cereb Cortex. 2014;24(7):1697–707. 23395849 10.1093/cercor/bht023

[44] Gratton C, Lee TG, Nomura EM, D’Esposito M. The effect of theta-burst TMS on cognitive control networks measured with resting state fMRI. Front Syst Neurosci. 2013;7:124. 24416003 10.3389/fnsys.2013.00124 PMC3874542

[45] Lee TG, D’Esposito M. The dynamic nature of top-down signals originating from prefrontal cortex: a combined fMRI-TMS study. J Neurosci. 2012;32(44):15458–66. 23115183 10.1523/JNEUROSCI.0627-12.2012 PMC3511853

[46] Abraham WC, Bear MF. Metaplasticity: the plasticity of synaptic plasticity. Trends Neurosci. 1996;19(4):126–30. 8658594 10.1016/s0166-2236(96)80018-x

[47] Huang YZ, Rothwell JC, Lu CS, Chuang WL, Lin WY, Chen RS. Reversal of plasticity-like effects in the human motor cortex. J Physiol. 2010;588(Pt 19):3683–93. 20660564 10.1113/jphysiol.2010.191361 PMC2997480

[48] Thickbroom GW. Transcranial magnetic stimulation and synaptic plasticity: experimental framework and human models. Exp Brain Res. 2007;180(4):583–93. 17562028 10.1007/s00221-007-0991-3

[49] Pasley BN, Allen EA, Freeman RD. State-dependent variability of neuronal responses to transcranial magnetic stimulation of the visual cortex. Neuron. 2009;62(2):291–303. 19409273 10.1016/j.neuron.2009.03.012 PMC2953477

[50] Fujiwara T, Rothwell JC. The after effects of motor cortex rTMS depend on the state of contraction when rTMS is applied. Clin Neurophysiol. 2004;115(7):1514–8. 15203052 10.1016/j.clinph.2004.01.021

[51] Ridding MC, Taylor JL, Rothwell JC. The effect of voluntary contraction on cortico-cortical inhibition in human motor cortex. J Physiol. 1995;487(Pt 2):541–8. 8558482 10.1113/jphysiol.1995.sp020898 PMC1156591

[52] Bestmann S, Swayne O, Blankenburg F, Ruff CC, Haggard P, Weiskopf N, Dorsal premotor cortex exerts state-dependent causal influences on activity in contralateral primary motor and dorsal premotor cortex. Cereb Cortex. 2008;18(6):1281–91. 17965128 10.1093/cercor/bhm159 PMC2600427

[53] Conte A, Gilio F, Iezzi E, Frasca V, Inghilleri M, Berardelli A. Attention influences the excitability of cortical motor areas in healthy humans. Exp Brain Res. 2007;182(1):109–17. 17516055 10.1007/s00221-007-0975-3

[54] Massimini M, Ferrarelli F, Huber R, Esser SK, Singh H, Tononi G. Breakdown of cortical effective connectivity during sleep. Science. 2005;309(5744):2228–32. 16195466 10.1126/science.1117256

[55] Hanlon CA, Dowdle LT, Austelle CW, DeVries W, Mithoefer O, Badran BW, What goes up, can come down: Novel brain stimulation paradigms may attenuate craving and craving-related neural circuitry in substance dependent individuals. Brain Res. 2015;1628(Pt A):199–209. 25770818 10.1016/j.brainres.2015.02.053 PMC4899830

[56] Narayana S, Zhang W, Rogers W, Strickland C, Franklin C, Lancaster JL, Concurrent TMS to the primary motor cortex augments slow motor learning. Neuroimage. 2014;85(Pt 3):971–84. 23867557 10.1016/j.neuroimage.2013.07.024 PMC4331120

[57] Sandrini M, Umilta C, Rusconi E. The use of transcranial magnetic stimulation in cognitive neuroscience: a new synthesis of methodological issues. Neurosci Biobehav Rev. 2011;35(3):516–36. 20599555 10.1016/j.neubiorev.2010.06.005

[58] Bestmann S, Ruff CC, Blankenburg F, Weiskopf N, Driver J, Rothwell JC. Mapping causal interregional influences with concurrent TMS-fMRI. Exp Brain Res. 2008;191(4):383–402. 18936922 10.1007/s00221-008-1601-8

[59] Silvanto J, Muggleton N, Walsh V. State-dependency in brain stimulation studies of perception and cognition. Trends Cogn Sci. 2008;12(12):447–54. 18951833 10.1016/j.tics.2008.09.004

[60] Cattaneo Z, Silvanto J, Pascual-Leone A, Battelli L. The role of the angular gyrus in the modulation of visuospatial attention by the mental number line. Neuroimage. 2009;44(2):563–8. 18848630 10.1016/j.neuroimage.2008.09.003 PMC2614466

[61] Cattaneo L, Sandrini M, Schwarzbach J. State-dependent TMS reveals a hierarchical representation of observed acts in the temporal, parietal, and premotor cortices. Cereb Cortex. 2010;20(9):2252–8. 20051360 10.1093/cercor/bhp291

[62] Silvanto J, Muggleton NG, Cowey A, Walsh V. Neural activation state determines behavioral susceptibility to modified theta burst transcranial magnetic stimulation. Eur J Neurosci. 2007;26(2):523–8. 17650122 10.1111/j.1460-9568.2007.05682.x

[63] Williams DS, Detre JA, Leigh JS, Koretsky AP. Magnetic resonance imaging of perfusion using spin inversion of arterial water. Proc Natl Acad Sci U S A. 1992;89(1):212–6. 1729691 10.1073/pnas.89.1.212 PMC48206

[64] Talagala SL, Noll DC. Functional MRI using steady-state arterial water labeling. Magn Reson Med. 1998;39(2):179–83. 9469699 10.1002/mrm.1910390203

[65] Edvinsson L, MacKenzie ET, McCulloch J. Cerebral Blood Flow and Metabolism. New York (US): Raven Press; 1993.

[66] Sokolof L Relation between physiological function and energy metabolism in the central nervous system. J Neurochem. 1977;29:13–26. 407330 10.1111/j.1471-4159.1977.tb03919.x

[67] Smith AM, Lewis BK, Ruttimann UE, Ye FQ, Sinnwell TM, Yang Y, Investigation of low frequency drift in fMRI signal. NeuroImage. 1999;9(5):526–33. 10329292 10.1006/nimg.1999.0435

[68] Bandettini PA, Wong EC, Hinks RS, Tikofsky RS, Hyde JS. Time course of EPI of human brain function during task activation. Magn Reson Med. 1992;25:390–7. 1614324 10.1002/mrm.1910250220

[69] Tjandra T, Brooks JC, Figueiredo P, Wise R, Matthews PM, Tracey I. Quantitative assessment of the reproducibility of functional activation measured with BOLD and MR perfusion imaging: implications for clinical trial design. NeuroImage. 2005;27(2):393–401. 15921936 10.1016/j.neuroimage.2005.04.021

[70] Wang J, Aguirre GK, Kimberg DY, Roc AC, Li L, Detre JA. Arterial spin labeling perfusion fMRI with very low task frequency. Magn Reson Med. 2003;49(5):796–802. 12704760 10.1002/mrm.10437

[71] Demeter E, Mirdamadi JL, Meehan SK, Taylor SF. Short theta burst stimulation to left frontal cortex prior to encoding enhances subsequent recognition memory. Cogn Affect Behav Neurosci. 2016;16(4):724–35. 27098772 10.3758/s13415-016-0426-3 PMC4955696

[72] Esslinger C, Schuler N, Sauer C, Gass D, Mier D, Braun U, Induction and quantification of prefrontal cortical network plasticity using 5 Hz rTMS and fMRI. Hum Brain Mapp. 2014;35(1):140–51. 22965696 10.1002/hbm.22165 PMC6868951

[73] Gaudeau-Bosma C, Moulier V, Allard AC, Sidhoumi D, Bouaziz N, Braha S, Effect of two weeks of rTMS on brain activity in healthy subjects during an *n*-back task: a randomized double blind study. Brain Stimul. 2013;6(4):569–75. 23194830 10.1016/j.brs.2012.10.009

[74] Guse B, Falkai P, Gruber O, Whalley H, Gibson L, Hasan A, The effect of long-term high frequency repetitive transcranial magnetic stimulation on working memory in schizophrenia and healthy controls--a randomized placebo-controlled, double-blind fMRI study. Behav Brain Res. 2013;237:300–7. 23022750 10.1016/j.bbr.2012.09.034

[75] Brunoni AR, Vanderhasselt MA. Working memory improvement with noninvasive brain stimulation of the dorsolateral prefrontal cortex: a systematic review and meta-analysis. Brain Cogn. 2014;86:1–9. 24514153 10.1016/j.bandc.2014.01.008

[76] Loo CK, Taylor JL, Gandevia SC, McDarmont BN, Mitchell PB, Sachdev PS. Transcranial magnetic stimulation (TMS) in controlled treatment studies: are some “sham” forms active? Biol Psychiatry. 2000;47(4):325–31. 10686267 10.1016/s0006-3223(99)00285-1

[77] Park DC, Polk TA, Park R, Minear M, Savage A, Smith MR. Aging reduces neural specialization in ventral visual cortex. Proc Natl Acad Sci U S A. 2004;101(35):13091–5. 15322270 10.1073/pnas.0405148101 PMC516469

[78] Carp J, Park J, Hebrank A, Park DC, Polk TA. Age-related neural dedifferentiation in the motor system. PLoS One. 2011;6(12):e29411. 22216274 10.1371/journal.pone.0029411 PMC3245287

[79] Sheehan DV, Lecrubier Y, Sheehan KH, Amorim P, Janavs J, Weiller E, The Mini-International Neuropsychiatric Interview (M.I.N.I.): the development and validation of a structured diagnostic psychiatric interview for DSM-IV and ICD-10. J Clin Psychiatry. 1998;59(Suppl 20):22–33;quiz 4–57. 9881538

[80] Rossi S, Hallett M, Rossini PM, Pascual-Leone A. Safety, ethical considerations, and application guidelines for the use of transcranial magnetic stimulation in clinical practice and research. Clin Neurophysiol. 2009;120(12):2008–39. 19833552 10.1016/j.clinph.2009.08.016 PMC3260536

[81] Beck AT, Steer RA, Brown GK. Manual for the Beck Depression Inventory-II. San Antonio (TX, US): Psychological Corporation; 1996.

[82] Ware J Jr., Kosinski M, Keller SD. A 12-Item Short-Form Health Survey: construction of scales and preliminary tests of reliability and validity. Med Care. 1996;34(3):220–33. 8628042 10.1097/00005650-199603000-00003

[83] Wilkinson GS. Wide Range Achievement Test 3 (WRAT3). Wilmington (DE, US): Wide Range, Inc.; 1993.

[84] Tombaugh TN. Trail Making Test A and B: normative data stratified by age and education. Arch Clin Neuropsychol. 2004;19(2):203–14. 15010086 10.1016/S0887-6177(03)00039-8

[85] Benson N, Hulac DM, Kranzler JH. Independent examination of the Wechsler Adult Intelligence Scale-Fourth Edition (WAIS-IV): what does the WAIS-IV measure? Psychol Assess. 2010;22(1):121–30. 20230158 10.1037/a0017767

[86] Taylor SF, Ho SS, Abagis T, Angstadt M, Maixner DF, Welsh RC, Changes in brain connectivity during a sham-controlled, transcranial magnetic stimulation trial for depression. J Affect Disord. 2018;232:143–51. 29494898 10.1016/j.jad.2018.02.019 PMC5858982

[87] Nystrom LE, Braver TS, Sabb FW, Delgado MR, Noll DC, Cohen JD. Working memory for letters, shapes, and locations: fMRI evidence against stimulusbased regional organization in human prefrontal cortex. Neuroimage. 2000;11(5 Pt 1):424–46. 10806029 10.1006/nimg.2000.0572

[88] Volle E, Kinkingnehun S, Pochon JB, Mondon K, Thiebaut de Schotten M, Seassau M, The functional architecture of the left posterior and lateral prefrontal cortex in humans. Cereb Cortex. 2008;18(10):2460–9. 18308710 10.1093/cercor/bhn010

[89] Smith EE, Jonides J. Neuroimaging analyses of human working memory. Proc Natl Acad Sci U S A. 1998;95(20):12061–8. 9751790 10.1073/pnas.95.20.12061 PMC21765

[90] Alsop DC, Detre JA, Golay X, Gunther M, Hendrikse J, Hernandez-Garcia L, Recommended implementation of arterial spin-labeled perfusion MRI for clinical applications: A consensus of the ISMRM perfusion study group and the European consortium for ASL in dementia. Magn Reson Med. 2015;73(1):102–16. 24715426 10.1002/mrm.25197 PMC4190138

[91] Nielsen JF, Hernandez-Garcia L. Functional perfusion imaging using pseudocontinuous arterial spin labeling with low-flip-angle segmented 3D spiral readouts. Magn Reson Med. 2013;69(2):382–90. 22488451 10.1002/mrm.24261

[92] Rossini PM, Burke D, Chen R, Cohen LG, Daskalakis Z, Di Iorio R, Noninvasive electrical and magnetic stimulation of the brain, spinal cord, roots and peripheral nerves: Basic principles and procedures for routine clinical and research application. An updated report from an I.F.C.N. Committee. Clin Neurophysiol. 2015;126(6):1071–107. 25797650 10.1016/j.clinph.2015.02.001 PMC6350257

[93] Oberman L, Edwards D, Eldaief M, Pascual-Leone A. Safety of theta burst transcranial magnetic stimulation: a systematic review of the literature. J Clin Neurophysiol. 2011;28(1):67–74. 21221011 10.1097/WNP.0b013e318205135f PMC3260517

[94] Mylius V, Ayache SS, Ahdab R, Farhat WH, Zouari HG, Belke M, Definition of DLPFC and M1 according to anatomical landmarks for navigated brain stimulation: inter-rater reliability, accuracy, and influence of gender and age. Neuroimage. 2013;78:224–32. 23567888 10.1016/j.neuroimage.2013.03.061

[95] Fitzgerald PB, Hoy K, McQueen S, Maller JJ, Herring S, Segrave R, A Randomized Trial of rTMS Targeted with MRI Based Neuro-Navigation in Treatment-Resistant Depression. Neuropsychopharmacology. 2009;34(5):1255–62. doi: 10.1038/npp.2008.233 19145228

[96] Worsley KJ, Friston KJ. Analysis of fMRI time-series revisited--again. Neuroimage. 1995;2(3):173–81. 9343600 10.1006/nimg.1995.1023

[97] Chumbley J, Worsley K, Flandin G, Friston K. Topological FDR for neuroimaging. NeuroImage. 2010;49(4):3057–64. 19944173 10.1016/j.neuroimage.2009.10.090 PMC3221040

[98] Friston KJ, Buechel C, Fink GR, Morris J, Rolls E, Dolan RJ. Psychophysiological and modulatory interactions in neuroimaging. Neuroimage. 1997;6(3):218–29. 9344826 10.1006/nimg.1997.0291

[99] Behzadi Y, Restom K, Liau J, Liu TT. A component based noise correction method (CompCor) for BOLD and perfusion based fMRI. Neuroimage. 2007;37(1):90–101. 17560126 10.1016/j.neuroimage.2007.04.042 PMC2214855

[100] Power JD, Schlaggar BL, Petersen SE. Recent progress and outstanding issues in motion correction in resting state fMRI. Neuroimage. 2015;105:536–51. 25462692 10.1016/j.neuroimage.2014.10.044 PMC4262543

[101] Pruim RHR, Mennes M, van Rooij D, Llera A, Buitelaar JK, Beckmann CF. ICAAROMA: A robust ICA-based strategy for removing motion artifacts from fMRI data. Neuroimage. 2015;112:267–77. 25770991 10.1016/j.neuroimage.2015.02.064

[102] Cole MW, Anticevic A, Repovs G, Barch D. Variable global dysconnectivity and individual differences in schizophrenia. Biol Psychiatry. 2011;70(1):43–50. 21496789 10.1016/j.biopsych.2011.02.010 PMC3204885

[103] Yang H, Long XY, Yang Y, Yan H, Zhu CZ, Zhou XP, Amplitude of low frequency fluctuation within visual areas revealed by resting-state functional MRI. Neuroimage. 2007;36(1):144–52. 17434757 10.1016/j.neuroimage.2007.01.054

[104] Tik M, Hoffmann A, Sladky R, Tomova L, Hummer A, Navarro de Lara L, Towards understanding rTMS mechanism of action: Stimulation of the DLPFC causes network-specific increase in functional connectivity. Neuroimage. 2017;162:289–96. 28912081 10.1016/j.neuroimage.2017.09.022

[105] Singh A, Erwin-Grabner T, Sutcliffe G, Paulus W, Dechent P, Antal A, Default mode network alterations after intermittent theta burst stimulation in healthy subjects. Transl Psychiatry. 2020;10(1):75. 32094326 10.1038/s41398-020-0754-5 PMC7040002

[106] Rounis E, Stephan KE, Lee L, Siebner HR, Pesenti A, Friston KJ, Acute changes in frontoparietal activity after repetitive transcranial magnetic stimulation over the dorsolateral prefrontal cortex in a cued reaction time task. J Neurosci. 2006;26(38):9629–38. 16988033 10.1523/JNEUROSCI.2657-06.2006 PMC6674444

[107] Vidal-Pineiro D, Martin-Trias P, Arenaza-Urquijo EM, Sala-Llonch R, Clemente IC, Mena-Sanchez I, Task-dependent activity and connectivity predict episodic memory network-based responses to brain stimulation in healthy aging. Brain Stimul. 2014;7(2):287–96. 24485466 10.1016/j.brs.2013.12.016 PMC4517193

[108] Tailby C, Masterton RA, Huang JY, Jackson GD, Abbott DF. Resting state functional connectivity changes induced by prior brain state are not network specific. Neuroimage. 2015;106:428–40. 25463462 10.1016/j.neuroimage.2014.11.037

[109] Plichta MM, Schwarz AJ, Grimm O, Morgen K, Mier D, Haddad L, Test-retest reliability of evoked BOLD signals from a cognitive-emotive fMRI test battery. NeuroImage. 2012;60(3):1746–58. 22330316 10.1016/j.neuroimage.2012.01.129

[110] Mumford JA, Nichols TE. Power calculation for group fMRI studies accounting for arbitrary design and temporal autocorrelation. Neuroimage. 2008;39(1):261–8. 17919925 10.1016/j.neuroimage.2007.07.061 PMC2423281

[111] Chen Y, Wang DJ, Detre JA. Test-retest reliability of arterial spin labeling with common labeling strategies. J Magn Reson Imaging. 2011;33(4):940–9. 21448961 10.1002/jmri.22345 PMC3069716

[112] Blumberger DM, Vila-Rodriguez F, Thorpe KE, Feffer K, Noda Y, Giacobbe P, Effectiveness of theta burst versus high-frequency repetitive transcranial magnetic stimulation in patients with depression (THREE-D): a randomised non-inferiority trial. Lancet. 2018;391(10131):1683–92. 29726344 10.1016/S0140-6736(18)30295-2

